# Developing consensus of evidence to target case finding surveys for podoconiosis: a potentially forgotten disease in India

**DOI:** 10.1093/trstmh/traa064

**Published:** 2020-11-09

**Authors:** Hope Simpson, K N Panicker, Leyanna Susan George, Jorge Cano, Melanie J Newport, Gail Davey, Kebede Deribe

**Affiliations:** Department of Disease Control, London School of Hygiene & Tropical Medicine, London, WC1E 7HT, UK; Deptartment of Community Medicine, Amrita Institute of Medical Sciences and Research Centre, Edappally, Kochi, Kerala, 682031, India; Deptartment of Community Medicine, Amrita Institute of Medical Sciences and Research Centre, Edappally, Kochi, Kerala, 682031, India; Department of Disease Control, London School of Hygiene & Tropical Medicine, London, WC1E 7HT, UK; Brighton and Sussex Centre for Global Health Research, Department of Global Health and Infection, Brighton and Sussex Medical School, Brighton, BN1 9PX, UK; Brighton and Sussex Centre for Global Health Research, Department of Global Health and Infection, Brighton and Sussex Medical School, Brighton, BN1 9PX, UK; School of Public Health, College of Health Sciences, Addis Ababa University, Addis Ababa, PO Box 9086, Ethiopia; Brighton and Sussex Centre for Global Health Research, Department of Global Health and Infection, Brighton and Sussex Medical School, Brighton, BN1 9PX, UK; School of Public Health, College of Health Sciences, Addis Ababa University, Addis Ababa, PO Box 9086, Ethiopia

**Keywords:** ecological niche modelling, evidence consensus, lymphedema, morbidity management and disability prevention, Podoconiosis, surveillance data, targeting surveys

## Abstract

**Background:**

Podoconiosis is a non-infectious geochemical lymphoedema of the lower legs associated with a significant burden of morbidity. There are historical reports of podoconiosis in India, but its current endemicity status is uncertain. In this investigation we aimed to prioritise the selection of districts for pilot mapping of podoconiosis in India.

**Methods:**

Through a consultative workshop bringing together expert opinion on podoconiosis with public health and NTDs in India, we developed a framework for the prioritisation of pilot areas. The four criteria for prioritisation were predicted environmental suitability for podoconiosis, higher relative poverty, occurrence of lymphoedema cases detected by the state health authorities and absence of morbidity management and disability prevention (MMDP) services provided by the National Programme for Elimination of Lymphatic Filariasis.

**Results:**

Environmental suitability for podoconiosis in India was predicted to be widespread, particularly in the mountainous east and hilly southwest of the country. Most of the districts with higher levels of poverty were in the central east and central west. Of 286 districts delineated by state representatives, lymphoedema was known to the health system in 189 districts and not recorded in 80. Information on MMDP services was unavailable for many districts, but 169 were known not to provide such services. We identified 35 districts across the country as high priority for mapping based on these criteria.

**Conclusions:**

Our results indicate widespread presence of conditions associated with podoconiosis in India, including areas with known lymphoedema cases and without MMDP services. This work is intended to support a rational approach to surveying for an unrecognised, geographically focal, chronic disease in India, with a view to scaling up to inform a national strategy if required.

## Introduction

Podoconiosis is a non-infectious geochemical lymphoedema of the lower legs, caused by long-term barefoot exposure to red clay soil of volcanic origin.^1,2^ The disease is associated with specific environmental and climatic factors and with cultural and behavioural practices that increase the risk of contact with irritant soils.^1^ The disease can be prevented by the use of footwear and the resulting lymphoedema is reversible in its early stages, while advanced lymphoedema can be managed to reduce the incidence of painful episodes of acute inflammatory attacks and prevent or slow progression.^3,4^ As such, there is a strong rationale for estimating the burden of disease and identifying populations at risk so that interventions can be scaled up and targeted to areas of need.

The global burden and distribution of podoconiosis are not precisely known: like other neglected tropical diseases (NTDs) associated with chronic morbidity, the disease is recognised to be grossly underdetected and underreported due to social, structural and epidemiological factors.^5^ Podoconiosis is a highly stigmatising condition, most prevalent in poor, rural communities with low access to healthcare for diagnosis and treatment. The disease is scarcely known among healthcare workers^6^ and has been considered ‘the most neglected tropical disease’.^7^ National policies and programmes targeting the disease are non-existent in most of the potentially endemic countries and organisations working on podoconiosis are limited to a few grassroots non-governmental organisations. Within this context, people affected by podoconiosis are unlikely to seek care; if they do, they are unlikely to be correctly diagnosed or reported.

Given the paucity of routine data on podoconiosis, population-based surveys combined with environmental modelling have become the mainstay of ongoing global efforts to estimate the burden and map the distribution of the disease.^8^ Surveys in Cameroon, Ethiopia and Rwanda have found a prevalence of between 0.06 and 4.05% at the national level, and higher within barefoot populations.^9–11^ Predictive models informed by empirical data from these surveys have revealed strong environmental associations, with the potential ecological niche mostly restricted to remote rural areas and characterised by annual precipitation levels and elevation and a lesser influence of vegetation, topography, hydrology and soil factors.^12^ Extrapolation of this niche across the African continent suggests that 114.5 million people in Africa live in areas suitable for the disease.^8^

The risk of podoconiosis depends also on the level of exposure to irritant soils—people who lack footwear and are engaged in occupations that involve extensive contact with soil, including farming, mining, and floor loom weaving, are at highest risk.^1^ In Ethiopia, sociodemographic risk factors for podoconiosis include lack of education, non-professional occupation and living in a house with mud or earth floors.^13^ Since these risk factors are also indicators of general poverty,^14^ we expect podoconiosis to be concentrated in deprived populations within environmentally suitable areas.

There is historical evidence of podoconiosis occurring in India,^2,15–17^ although cases are not currently reported by the health system. The application of an evidence consensus framework, a method designed to evaluate the evidence for the occurrence of a disease based on multiple weighted criteria,^18,19^ identified strong evidence of podoconiosis occurrence in India.^20^ The evidence consensus framework took account of cases reported in published and grey literature, as well as likely causes of underreporting, including the occurrence of diseases with clinically similar presentations that might mask the incidence of podoconiosis. Despite strong evidence for podoconiosis in India, its current endemicity status is unknown. The disease may have been eliminated through socio-economic development, or it may persist in suitable environments and populations, unrecognised by the health system due to underdetection or misdiagnosis.

Lymphoedema is certainly widespread in India,^21^ which bears one of the highest burdens of lymphatic filariasis (LF) globally,^22,23^ with 600 million estimated to be at risk of the disease and 800 000 estimated cases of lymphoedema.^24,25^ Filarial and geochemical lymphoedema show substantial clinical overlap and are both associated with acute attacks, which are painful for patients and cause further lymphatic impairment, leading to worsening of the condition.^26^ Podoconiosis surveys in Africa have shown that podoconiosis is frequently misdiagnosed as LF, the latter being more widely recognised by healthcare workers.^11^ This not only risks underestimation of the burden of podoconiosis, but may also confound the measurement of progress towards LF elimination.

From the perspective of case management for lymphoedema, the distinction of the cause is less important: all patients require morbidity management and disability prevention (MMDP), including frequent washing, elevation and massage, treatment of secondary infections and management of acute attacks to prevent further lymphatic impairment.^4^ In India, training on self-care is provided through the National Programme to Eliminate Lymphatic Filariasis (NPELF), under the National Vector Borne Disease Control Programme (NVBDCP).^25^ This implies that hypothetically, podoconiosis cases occurring within LF-endemic districts may benefit from MMDP if detected through routine channels for LF morbidity case finding. In contrast, cases of lymphoedema arising in non-LF-endemic districts are unlikely to receive MMDP through the NPELF. With this in mind, case finding activities for podoconiosis would be of most benefit to patients if targeted to districts not currently providing MMDP services through the NPELF.

In this investigation we aimed to prioritise the selection of districts for pilot mapping of podoconiosis in India according to four criteria: potential environmental suitability for podoconiosis, higher relative levels of poverty (assuming lower access to footwear and thus higher exposure to irritant soils among the poorest), occurrence of lymphoedema cases detected by the state health authorities and the absence of MMDP services provided by the LF programme. This is intended to inform a rational approach to surveying for an unrecognised, geographically focal, chronic disease in a vast and varied country, with a view to scaling up to inform a national strategy if required.

## Methods

### Study design

This was a consensus development exercise, applying a systematic framework to consolidate expert opinion and programmatic experience from within India with empirical evidence from other countries.

### Study location

India is a South Asian country with a population of >1.3 billion and a total land area of >3 287 263 km^2^.^27^ It is organised into 28 administrative states and 8 union territories,^28^ further divided into districts, totalling 668 in 2015.^29^ State governments are responsible for the provision of healthcare and the public health system, while certain specific health programmes and initiatives are organised by the central government.^30,31^

### LF programme and MMDP for lymphoedema

Government-led programmes to control LF in India have been implemented for many years, with the current NPELF in place since 2004.^32^ Its key strategic pillars are the interruption of transmission through mass drug administration (MDA) and the alleviation of suffering through MMDP. The programme initially covered 202 districts in 20 states and union territories and was subsequently scaled up to reach 256 endemic districts targeting a population of about 600 million.^32^ During MDA campaigns, cases of lymphoedema are recorded at the village or subcentre level through house-to-house visits. Cases are aggregated at the primary health centre (PHC), district and state levels. People with lymphoedema are given demonstrations and training on World Health Organization–recommended hygiene-based management of lymphoedema and are encouraged to practise self-care.^32^

### Development of the consensus framework

The consensus framework for the prioritisation of districts for piloting podoconiosis surveys was developed through a consultative workshop held at the Amrita Institute of Medical Sciences Ernakulam, Kerala, 10-11 December 2019. Experts in public health, community medicine, NTDs and LF from all states and union territories in India (hereafter ‘state representatives’) and international experts on podoconiosis were invited to this workshop in order to share their expertise for development of the framework. Those who were unable to join were engaged through remote communication after the workshop.

**Figure 1.**
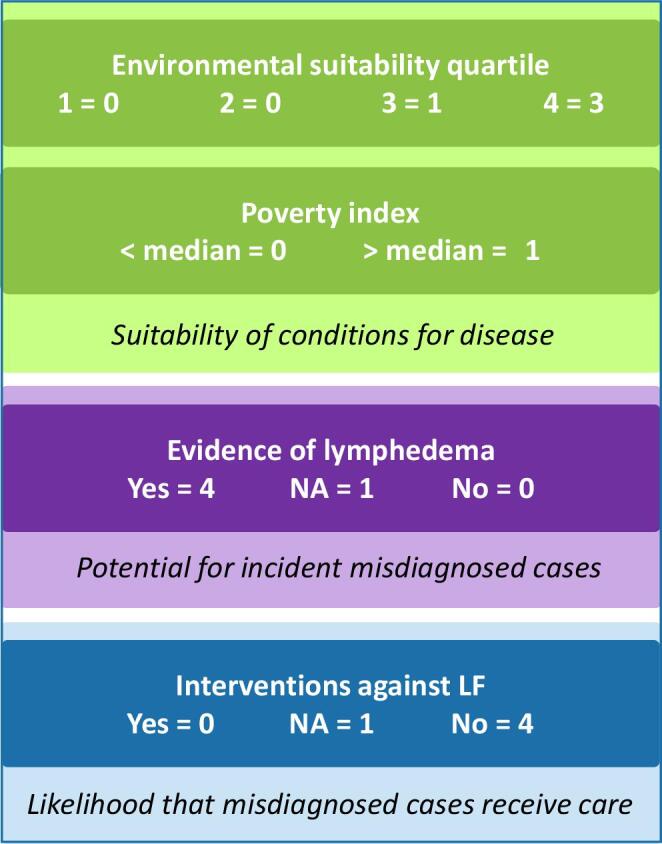
Weighted criteria for prioritisation of districts for pilot mapping of podoconiosis in India.

Following presentations on the clinical and epidemiological aspects of podoconiosis, its treatment, geographic distribution and environmental associations and LF in India, the group discussed and refined the framework to consolidate evidence that would determine priority selection of districts for pilot mapping. It was agreed that the framework should prioritise districts with suitable environmental conditions for podoconiosis, where the population was most at risk based on socio-economic indicators of poverty, where lymphoedema cases were known to the health system and where patients were less likely to be served by MMDP services (Figure 1).

When the final framework was agreed upon, state representatives formed groups to discuss the target criteria in each district within their states. On the final day of the workshop, state representatives presented the results of the consensus framework to grade the priority for mapping podoconiosis and any data gaps in each district. Data gaps were later filled through remote consultation with state health officials.

The final criteria for targeting pilot mapping surveys were district predicted to be suitable or moderately suitable for podoconiosis, district poverty higher than the median, lymphoedema cases known to the health system within the district and district does not currently implement MDA against LF and transmission interruption not recently certified.

### Data sources

Environmental suitability for podoconiosis was extrapolated from an ensemble model using podoconiosis occurrence data from eight countries in Africa, primarily from national surveys in Cameroon, Ethiopia and Rwanda,^9,10,13^ and a suite of environmental covariates potentially associated with the disease. The data sources and development of this model have been described elsewhere.^8^ Elevation and annual precipitation were the strongest predictors within the model, with the highest suitability predicted in areas with 1000– 1500 mm annual precipitation and elevation >1000 m above sea level. Other environmental predictors included soil characteristics (clay and silt fractions) and soil acidity of the topsoil, the mean land surface temperature, distance to the nearest body of water and enhanced vegetation index, a measure of vegetation cover. The mean suitability was projected at a resolution of grid cells of 5 km × 5 km and categorised into quartiles. The modal quartile of averaged suitability was calculated in each district. Districts with a modal quartile of 4 were classified as ‘suitable’, those with a modal quartile of 3 were classified as ‘moderately suitable’ and those with a modal quartile <3 were classified as ‘not suitable’.

We used a multidimensional index of poverty (MDPI) produced by the Oxford Poverty and Human Development Initiative^14^ to classify relative levels of poverty at the district level. The MDPI includes various indicators of health, education and living standards and takes account of the proportion of the population who are poor and the intensity of deprivation among the poor.^14^ The district-level MDPI was assigned to each district defined by the GADM 2015 based on state and district names, using fuzzy logic implemented in R (R version 4.0.1 (2020-06-06), R Foundation, Vienna, Austria) to allow for variation in spellings. Districts with an MDPI above the median value were categorised as ‘more deprived’.

State representatives compiled surveillance data on the incidence of lymphoedema detected through the health system in each district in their own states. Using these data, each district was categorised according to the known occurrence of lymphoedema: ‘present’, ‘not detected’ or ‘unknown’.

The state representatives also contributed programmatic information on the implementation of interventions against LF through the NPELF in each district. Districts classified as endemic or in which interruption of LF transmission had recently been certified were considered the most likely to deliver MMDP services for lymphoedema patients.

### Data analysis

The units of analysis were districts defined by the state representatives. Most of these districts were represented in the dataset of second-level administrative areas in India defined by the database of Global Administrative Areas (GADM) in 2015,^29^ while some were represented in the equivalent version of the dataset from 2012^28^ but had been redistricted prior to 2015. The districts described by state representatives were linked to the districts defined by the GADM 2015 and the GADM 2012 using fuzzy logic, as described above. Districts that were not represented within either GADM dataset were manually linked by state representatives to districts from the 2015 shapefile.

The evidence was collated through a scoring system that attributed fixed scores to different levels of each of the target criteria (Figure 1). The component scores assigned to each district were summed to provide an overall consensus score. Districts scoring >75% of the maximum score were considered high priority for mapping.

In order to map the results, the evidence compiled in the workshop was linked to the shapefile of districts in 2015. Full details of the linkage of districts to the shapefile are provided in Supplementary Methods.

## Results

Representatives from 27 states compiled data for 286 districts. The continuous extrapolated environmental suitability for podoconiosis in India is shown in Supplementary Figure 1. The modal quartile of averaged suitability was calculated in each district and linked to the district cartography. In total, 101 of 286 districts described by the state representatives and 191 of 668 from the GADM 2015 shapefile were predicted to have high suitability for podoconiosis (Table 1). Moderate suitability was predicted in 100 districts defined by the state representatives and 190 from the GADM 2015 shapefile. Twenty-three states and union territories included districts from the GADM 2015 shapefile that were predicted highly suitable.

**Table 1.** Criteria for prioritising pilot mapping of podoconiosis in districts of India

	Levels of criterion		
Criteria for mapping	Number of districts in each category (N=286)		
Environmental suitability	High (MQ4)	Moderate (MQ3)	Low (MQ1 and 2)
	101	100	85
Relative poverty	Higher	Lower	
	124	162	
Evidence of lymphoedema	Recorded	Not recorded	Information NA
	189	80	17
MMDP services	Implemented	Not implemented	Information NA
	15	169	102

Numbers of districts are those described by state representatives. MQ: modal quartile (see Methods)

Data on the incidence of lymphoedema was obtained for 269 districts within 24 states. The state representatives reported lymphoedema cases known to the health system in 189 districts. Information on MDA implementation was available for 184 districts, representing 19 states. These data indicated that 15 districts were LF endemic or had recently interrupted transmission, while 169 had no LF programme coverage and were thus unlikely to be implementing MMDP services (Table 1).

**Figure 2.**
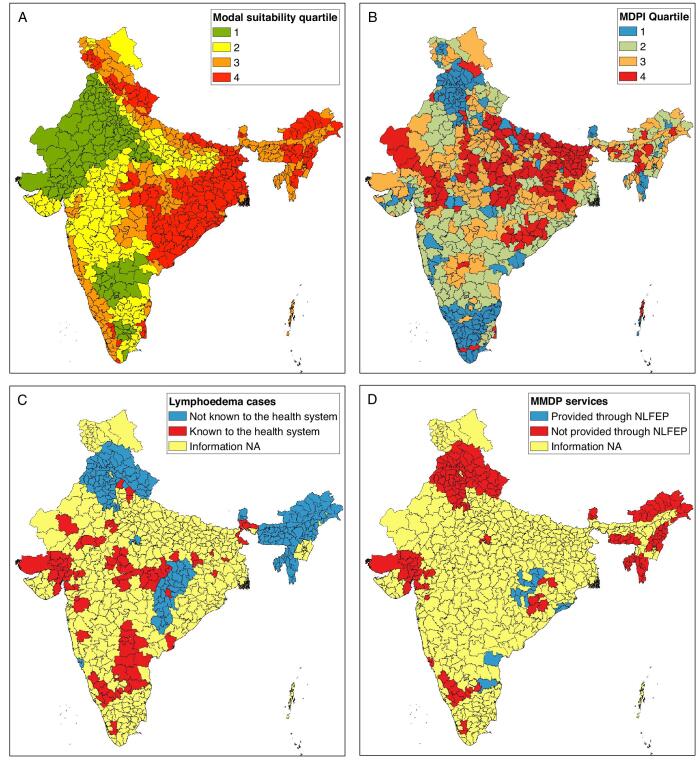
Component scores for prioritisation of podoconiosis mapping surveys at the district level.

**Figure 3.**
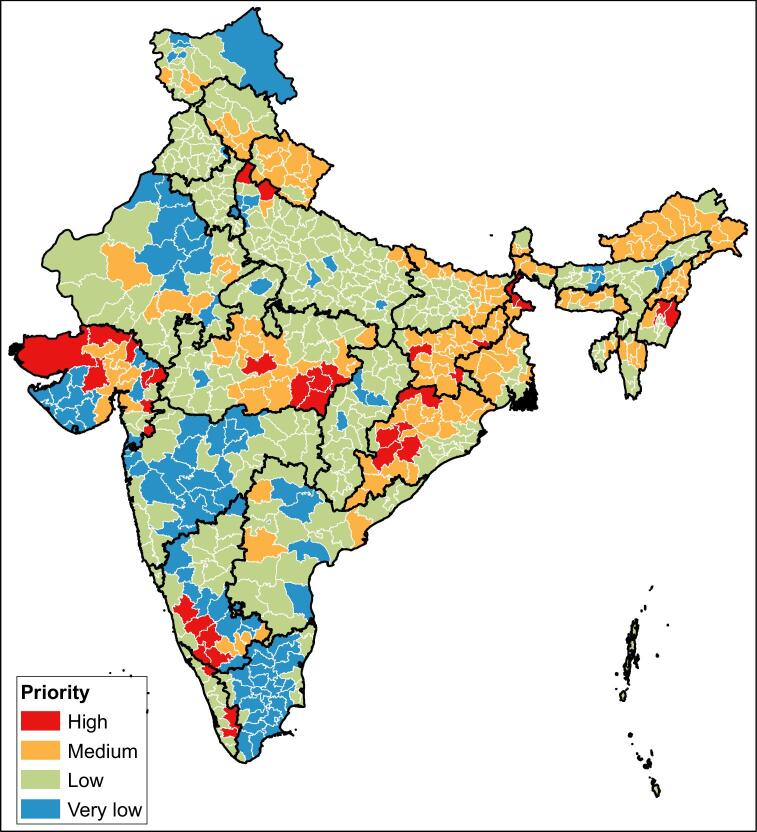
Level of priority for mapping surveys for podoconiosis at district level in India.

Figure 2 shows the levels of each component at the district level. Supplementary Table 1 shows the full results of the weighted scoring system for all of the districts identified by the state representatives. A full summary of the evidence categories assigned for all observed configurations of component scores is shown in Supplementary Figure 2. In total, 35 districts were identified as high priority for mapping and 108 were classified as medium priority (Table 2, Figure 3).

**Table 2.** Total numbers of districts by level of priority for mapping

Evidence score (%)	Priority	Number of districts
75–100	High	35
50–74	Medium	108
25–49	Low	134
0–24	Very low	9

The districts listed by state representatives that were predicted highly suitable represented 17 states and union territories. Districts with higher levels of poverty were in 19 states and union territories. Lymphoedema cases were known to the health system in 12 states and union territories and 17 states and union territories were not known to implement interventions against LF.

## Discussion

Through a cooperative, consultative process, we have developed and applied an evidence-based framework to prioritise the selection of districts for podoconiosis case finding surveys in India. The key criteria identified through the consensus development process were suitability for podoconiosis based on evidence from environmental modelling and socio-economic indicators, the occurrence of conditions clinically consistent with the disease according to local expert opinion and the absence of case management services based on the coverage of the NPELF. This enabled the identification of 35 districts where the disease was most likely to occur and where patients were least likely to be able to access MMDP services. These districts are considered to be key targets for initial surveys to establish the endemicity status of podoconiosis in India.

The priority districts we identified are dispersed through nine states across India. None of the districts were assigned the maximum score across all of the criteria, and among those identified as being high priority mapping targets, there is variability in their suitability against different criteria. Those with the highest scores had known cases of lymphoedema and no known MMDP services but were predicted to be only moderately environmentally suitable and showed lower rates of relative poverty. Other districts identified as high priority had high environmental suitability, known cases of lymphoedema and no information on MMDP services. The framework and results are intended to provide an evidence-based tool to facilitate and inform decisions rather than to drive them. Other criteria, such as logistical feasibility of surveying, will also be considered when these decisions are made.

A key strength of this exercise was its success in consolidating a substantive knowledge base from experts of multiple relevant disciplines across most states of India. The consultative workshop enabled the sharing of knowledge and ideas among a group with a great diversity of experience and brought a varied range of perspectives to the development of the consensus framework. The outcome was a locally relevant evidence base supported by varied sources of empirical data and expert opinion. The collaborative process also built a supportive and knowledgeable local network that will be vital to the success of future efforts to map and address the burden of podoconiosis in India, if it is found to be endemic.

Throughout the consultation, there was ongoing discussion on the justification for each of the criteria within the framework. There was recognition of the need to balance rational resource allocation with sensitivity to detect a disease that might occur at very low prevalence, if at all, in a very large geographical area. Due to the lack of contemporary data on podoconiosis in India, suitability for podoconiosis was extrapolated from an environmental model informed by data from Africa. It is not known whether the environmental associations of podoconiosis in Africa can be applied in India, but since podoconiosis has strong environmental drivers and is associated with specific geographic and climatic conditions, the main environmental associations are expected to be consistent across different geographical areas. This is supported by experience of podoconiosis surveys in Cameroon that identified the highest rates of the disease in areas predicted to be highly suitable by a model based mainly on data from Ethiopia.^9^ In this investigation, districts were classified as highly suitable if most of the area within them was in the upper quartile of suitability based on environmental model predictions. This classification may have deprioritised districts with varied environmental conditions and focal suitability for podoconiosis. Prospective pilot surveys in India will provide an opportunity to evaluate the external validity of the existing models. Furthermore, any newly identified cases will be used to develop more specific models of environmental suitability within India, which will inform the scale-up of mapping surveys and burden estimation.

The investigation was affected by missing data, particularly on the occurrence of lymphoedema and the provision of MMDP services at the district level. The true distribution of lymphoedema in India, which may include cases of podoconiosis, is likely to be broader than that represented by existing surveillance data. This may have led to deprioritisation of potentially endemic districts lacking data. The coverage of MMDP services may also be broader than we estimated, since such services may be delivered outside of the LF elimination programme or at a small local scale. We do not consider this to be a significant limitation to the work: if surveys are implemented in districts where MMDP is already provided, it may be possible to strengthen and support these services to ensure they reach all people affected by lymphoedema.

Our results will help determine the contemporary endemicity of podoconiosis in India, refine global understanding of the epidemiology of the disease and guide future mapping strategies. We recommend a pilot study using robust sampling and diagnostic strategies be conducted in one or two districts. The aims of this study will be to establish the occurrence of podoconiosis and to investigate its social and spatial epidemiology in India. The study must be carefully designed to detect spatial and environmental variation, which are critical for future modelling of the risk of podoconiosis across India.

## Conclusion

The consensus development framework we have applied constitutes an important first step in building the evidence for podoconiosis endemicity in a country where there is a strong indication of disease existence but scarce data for public health action. As a preliminary exercise, this analysis suggests that podoconiosis may occur in multiple districts across India. If true, this implies a large population at risk, some of whom would not be covered by existing services for MMDP. Case searches for podoconiosis should be planned in districts most likely to harbour cases of podoconiosis and least likely to provide MMDP to those affected. These targeted searches will help to clarify the epidemiological status of podoconiosis in India, supporting the global understanding of the burden of podoconiosis and efforts to ensure access to prevention and treatment for those at risk of or affected by the disease.

## Supplementary Material

traa064_Supplemental_Files
