# Supplementary material for: Developing consensus of evidence to target case finding surveys for podoconiosis: a potentially forgotten disease in India
Source: Trans R Soc Trop Med Hyg. 2020 Nov 9;114(12):908–15. doi: 10.1093/trstmh/traa064 (PMC7738658; doi:10.1093/trstmh/traa064)
Supplement: traa064_Supplemental_Files [file traa064_supplemental_files.zip › Supplementary_Table_Evidence_Scores.docx]

Supplementary Table 1: Scores Assigned through the Evidence Consensus Framework for the Prioritisation of Districts for Pilot Mapping of Podoconiosis in India.

| **State** | **District** | **Environmental suitability^1^** | | **Poverty^2^** | | **Lymphoedema^3^** | | **MMDP services^4^** | | **Consensus score^5^** | **Percentage** | **Priority** |
| --- | --- | --- | --- | --- | --- | --- | --- | --- | --- | --- | --- | --- |
|  |  | **Evidence** | **Score** | **Evidence** | **Score** | **Evidence** | **Score** | **Evidence** | **Score** |  |  |  |
| Uttar Pradesh | Bijnor | **3** | 1 | **3** | 1 | **1** | 4 | **0** | 4 | 10 | 83.3 | High |
| Gujarat | Narmada | **3** | 1 | **4** | 1 | **1** | 4 | **0** | 4 | 10 | 83.3 | High |
| Gujarat | The Dangs | **3** | 1 | **4** | 1 | **1** | 4 | **0** | 4 | 10 | 83.3 | High |
| Kerala | Pathanamthita | **3** | 1 | **1** | 0 | **1** | 4 | **0** | 4 | 9 | 75 | High |
| Madra Pradesh | Balaghat | **4** | 3 | **4** | 1 | **1** | 4 | **NA** | 1 | 9 | 75 | High |
| Gujarat | Banaskatha | **1** | 0 | **3** | 1 | **1** | 4 | **0** | 4 | 9 | 75 | High |
| Karnataka | Chikkamagaluru | **3** | 1 | **2** | 0 | **1** | 4 | **0** | 4 | 9 | 75 | High |
| Gujarat | Dahod | **2** | 0 | **4** | 1 | **1** | 4 | **0** | 4 | 9 | 75 | High |
| West Bengal | Dakshin Dinajpur | **4** | 3 | **3** | 1 | **1** | 4 | **NA** | 1 | 9 | 75 | High |
| Madra Pradesh | Dindori | **4** | 3 | **4** | 1 | **1** | 4 | **NA** | 1 | 9 | 75 | High |
| Karnataka | Hassan | **3** | 1 | **1** | 0 | **1** | 4 | **0** | 4 | 9 | 75 | High |
| Kerala | Iddukki | **3** | 1 | **1** | 0 | **1** | 4 | **0** | 4 | 9 | 75 | High |
| Jharkand | Jamtara | **4** | 3 | **4** | 1 | **1** | 4 | **NA** | 1 | 9 | 75 | High |
| Gujarat | Kutch | **1** | 0 | **3** | 1 | **1** | 4 | **0** | 4 | 9 | 75 | High |
| Odisha | Kalahandi | **4** | 3 | **4** | 1 | **NA** | 1 | **0** | 4 | 9 | 75 | High |
| Odisha | Kandhamal | **4** | 3 | **4** | 1 | **NA** | 1 | **0** | 4 | 9 | 75 | High |
| Karnataka | Kodagu | **3** | 1 | **1** | 0 | **1** | 4 | **0** | 4 | 9 | 75 | High |
| Jharkand | Latehar | **4** | 3 | **4** | 1 | **1** | 4 | **NA** | 1 | 9 | 75 | High |
| Madra Pradesh | Mandla | **4** | 3 | **4** | 1 | **1** | 4 | **NA** | 1 | 9 | 75 | High |
| Karnataka | Mysuru | **3** | 1 | **1** | 0 | **1** | 4 | **0** | 4 | 9 | 75 | High |
| Gujarat | Panchmahal | **2** | 0 | **4** | 1 | **1** | 4 | **0** | 4 | 9 | 75 | High |
| Madra Pradesh | Raisen | **4** | 3 | **3** | 1 | **1** | 4 | **NA** | 1 | 9 | 75 | High |
| Gujarat | Sabarkantha | **1** | 0 | **3** | 1 | **1** | 4 | **0** | 4 | 9 | 75 | High |
| Uttar Pradesh | Shaharanpur | **2** | 0 | **3** | 1 | **1** | 4 | **0** | 4 | 9 | 75 | High |
| Jharkand | Saraikela | **4** | 3 | **4** | 1 | **1** | 4 | **NA** | 1 | 9 | 75 | High |
| Manipur | Senapati | **4** | 3 | **3** | 1 | **NA** | 1 | **0** | 4 | 9 | 75 | High |
| Madra Pradesh | Seoni | **4** | 3 | **4** | 1 | **1** | 4 | **NA** | 1 | 9 | 75 | High |
| Karnataka | Shivamogga | **3** | 1 | **2** | 0 | **1** | 4 | **0** | 4 | 9 | 75 | High |
| Odisha | Sundergarh | **4** | 3 | **3** | 1 | **NA** | 1 | **0** | 4 | 9 | 75 | High |
| Gujarat | Surendranagar | **1** | 0 | **3** | 1 | **1** | 4 | **0** | 4 | 9 | 75 | High |
| Manipur | Ukhrul | **4** | 3 | **3** | 1 | **NA** | 1 | **0** | 4 | 9 | 75 | High |
| West Bengal | Uttar Dinajpur | **4** | 3 | **4** | 1 | **1** | 4 | **NA** | 1 | 9 | 75 | High |
| Kerala | Wayanadu | **3** | 1 | **1** | 0 | **1** | 4 | **0** | 4 | 9 | 75 | High |
| Odisha | Balangir | **4** | 3 | **3** | 1 | **NA** | 1 | **0** | 4 | 9 | 75 | High |
| Odisha | Sonepur | **4** | 3 | **3** | 1 | **NA** | 1 | **0** | 4 | 9 | 75 | High |
| Gujarat | Ahmedabad | **1** | 0 | **1** | 0 | **1** | 4 | **0** | 4 | 8 | 66.7 | Medium |
| Gujarat | Anand | **1** | 0 | **2** | 0 | **1** | 4 | **0** | 4 | 8 | 66.7 | Medium |
| Karnataka | Bangalore Urban | **2** | 0 | **1** | 0 | **1** | 4 | **0** | 4 | 8 | 66.7 | Medium |
| Gujarat | Bharuch | **2** | 0 | **2** | 0 | **1** | 4 | **0** | 4 | 8 | 66.7 | Medium |
| Gujarat | Bhavnagar | **2** | 0 | **2** | 0 | **1** | 4 | **0** | 4 | 8 | 66.7 | Medium |
| Himachal Pradesh | Bilaspur | **4** | 3 | **3** | 1 | **0** | 0 | **0** | 4 | 8 | 66.7 | Medium |
| Arunachal Pradesh | Changlang | **4** | 3 | **3** | 1 | **0** | 0 | **0** | 4 | 8 | 66.7 | Medium |
| Andra Pradesh | East Godhavari | **4** | 3 | **1** | 0 | **1** | 4 | **NA** | 1 | 8 | 66.7 | Medium |
| Arunachal Pradesh | East Kameng | **4** | 3 | **4** | 1 | **0** | 0 | **0** | 4 | 8 | 66.7 | Medium |
| Gujarat | Gandhinagar | **1** | 0 | **2** | 0 | **1** | 4 | **0** | 4 | 8 | 66.7 | Medium |
| Madra Pradesh | Jabalpur | **4** | 3 | **2** | 0 | **1** | 4 | **NA** | 1 | 8 | 66.7 | Medium |
| Mehalaya | East Jaintia Hills | **4** | 3 | **4** | 1 | **0** | 0 | **0** | 4 | 8 | 66.7 | Medium |
| Mehalaya | West Jaintia Hills | **4** | 3 | **4** | 1 | **0** | 0 | **0** | 4 | 8 | 66.7 | Medium |
| Gujarat | Kheda | **1** | 0 | **2** | 0 | **1** | 4 | **0** | 4 | 8 | 66.7 | Medium |
| Nagaland | Kiphire | **4** | 3 | **3** | 1 | **0** | 0 | **0** | 4 | 8 | 66.7 | Medium |
| Karnataka | Kolar | **2** | 0 | **2** | 0 | **1** | 4 | **0** | 4 | 8 | 66.7 | Medium |
| Arunachal Pradesh | Kurung Kumey | **4** | 3 | **3** | 1 | **0** | 0 | **0** | 4 | 8 | 66.7 | Medium |
| Gujarat | Mehsana | **1** | 0 | **2** | 0 | **1** | 4 | **0** | 4 | 8 | 66.7 | Medium |
| Karnataka | Mandya | **2** | 0 | **1** | 0 | **1** | 4 | **0** | 4 | 8 | 66.7 | Medium |
| Gujarat | Patan | **1** | 0 | **2** | 0 | **1** | 4 | **0** | 4 | 8 | 66.7 | Medium |
| Karnataka | Bangaluru (R) | **2** | 0 | **1** | 0 | **1** | 4 | **0** | 4 | 8 | 66.7 | Medium |
| Nagaland | Tuensang | **4** | 3 | **3** | 1 | **0** | 0 | **0** | 4 | 8 | 66.7 | Medium |
| Arunachal Pradesh | Upper Subansiri | **4** | 3 | **3** | 1 | **0** | 0 | **0** | 4 | 8 | 66.7 | Medium |
| Uttarakhand | Pauri Garhwal | **4** | 3 | **2** | 0 | **0** | 0 | **0** | 4 | 7 | 58.3 | Medium |
| Uttarakhand | Haridwar | **4** | 3 | **2** | 0 | **0** | 0 | **0** | 4 | 7 | 58.3 | Medium |
| Mizoram | Aizawl | **4** | 3 | **1** | 0 | **0** | 0 | **0** | 4 | 7 | 58.3 | Medium |
| West Bengal | Alipurduar | **3** | 1 | **3** | 1 | **1** | 4 | **NA** | 1 | 7 | 58.3 | Medium |
| Uttarakhand | Almora | **4** | 3 | **2** | 0 | **0** | 0 | **0** | 4 | 7 | 58.3 | Medium |
| Arunachal Pradesh | Anjaw | **4** | 3 | **2** | 0 | **0** | 0 | **0** | 4 | 7 | 58.3 | Medium |
| Madra Pradesh | Anuppur | **3** | 1 | **4** | 1 | **1** | 4 | **NA** | 1 | 7 | 58.3 | Medium |
| Uttarakhand | Bageshwar | **4** | 3 | **2** | 0 | **0** | 0 | **0** | 4 | 7 | 58.3 | Medium |
| Madra Pradesh | betul | **3** | 1 | **3** | 1 | **1** | 4 | **NA** | 1 | 7 | 58.3 | Medium |
| Uttarakhand | Chamoli | **4** | 3 | **2** | 0 | **0** | 0 | **0** | 4 | 7 | 58.3 | Medium |
| Uttarakhand | Champawa | **4** | 3 | **2** | 0 | **0** | 0 | **0** | 4 | 7 | 58.3 | Medium |
| Mizoram | Champhai | **4** | 3 | **1** | 0 | **0** | 0 | **0** | 4 | 7 | 58.3 | Medium |
| Nagaland | Dimapur | **4** | 3 | **2** | 0 | **0** | 0 | **0** | 4 | 7 | 58.3 | Medium |
| Arunachal Pradesh | Pasighat | **4** | 3 | **1** | 0 | **0** | 0 | **0** | 4 | 7 | 58.3 | Medium |
| Sikkim | East Sikkim | **4** | 3 | **1** | 0 | **0** | 0 | **0** | 4 | 7 | 58.3 | Medium |
| Himachal Pradesh | Hamirpur | **4** | 3 | **1** | 0 | **0** | 0 | **0** | 4 | 7 | 58.3 | Medium |
| West Bengal | Jalpaiguri | **3** | 1 | **3** | 1 | **1** | 4 | **NA** | 1 | 7 | 58.3 | Medium |
| Jharkand | Kodema | **3** | 1 | **3** | 1 | **1** | 4 | **NA** | 1 | 7 | 58.3 | Medium |
| Nagaland | Kohima | **4** | 3 | **1** | 0 | **0** | 0 | **0** | 4 | 7 | 58.3 | Medium |
| Arunachal Pradesh | Lower Dibang Valley | **4** | 3 | **2** | 0 | **0** | 0 | **0** | 4 | 7 | 58.3 | Medium |
| Arunachal Pradesh | Lower Subansiri | **4** | 3 | **2** | 0 | **0** | 0 | **0** | 4 | 7 | 58.3 | Medium |
| Himachal Pradesh | Mandi | **4** | 3 | **1** | 0 | **0** | 0 | **0** | 4 | 7 | 58.3 | Medium |
| Nagaland | Mokokchung | **4** | 3 | **1** | 0 | **0** | 0 | **0** | 4 | 7 | 58.3 | Medium |
| Madra Pradesh | Narsinghpur | **3** | 1 | **3** | 1 | **1** | 4 | **NA** | 1 | 7 | 58.3 | Medium |
| Jharkand | Palamu | **3** | 1 | **4** | 1 | **1** | 4 | **NA** | 1 | 7 | 58.3 | Medium |
| Haryana | Panchkula | **4** | 3 | **1** | 0 | **0** | 0 | **0** | 4 | 7 | 58.3 | Medium |
| Arunachal Pradesh | Papum Pare | **4** | 3 | **2** | 0 | **0** | 0 | **0** | 4 | 7 | 58.3 | Medium |
| Nagaland | Peren | **4** | 3 | **2** | 0 | **0** | 0 | **0** | 4 | 7 | 58.3 | Medium |
| Nagaland | Phek | **4** | 3 | **2** | 0 | **0** | 0 | **0** | 4 | 7 | 58.3 | Medium |
| Uttarakhand | Pithoragarh | **4** | 3 | **2** | 0 | **0** | 0 | **0** | 4 | 7 | 58.3 | Medium |
| Odisha | Raigarha | **3** | 1 | **3** | 1 | **NA** | 1 | **0** | 4 | 7 | 58.3 | Medium |
| Uttarakhand | Rudrapraya | **4** | 3 | **2** | 0 | **0** | 0 | **0** | 4 | 7 | 58.3 | Medium |
| Mizoram | Serchhip | **4** | 3 | **1** | 0 | **0** | 0 | **0** | 4 | 7 | 58.3 | Medium |
| Madra Pradesh | Shahdol | **3** | 1 | **4** | 1 | **1** | 4 | **NA** | 1 | 7 | 58.3 | Medium |
| Himachal Pradesh | Shimla | **4** | 3 | **1** | 0 | **0** | 0 | **0** | 4 | 7 | 58.3 | Medium |
| Madra Pradesh | Sidhi | **3** | 1 | **4** | 1 | **1** | 4 | **NA** | 1 | 7 | 58.3 | Medium |
| Himachal Pradesh | Sirmaur | **4** | 3 | **2** | 0 | **0** | 0 | **0** | 4 | 7 | 58.3 | Medium |
| Himachal Pradesh | Solan | **4** | 3 | **1** | 0 | **0** | 0 | **0** | 4 | 7 | 58.3 | Medium |
| Sikkim | South Sikkim | **4** | 3 | **1** | 0 | **0** | 0 | **0** | 4 | 7 | 58.3 | Medium |
| Manipur | Tamenglong | **3** | 1 | **3** | 1 | **NA** | 1 | **0** | 4 | 7 | 58.3 | Medium |
| Uttarakhand | Tehri Garhwal | **4** | 3 | **2** | 0 | **0** | 0 | **0** | 4 | 7 | 58.3 | Medium |
| Uttarakhand | Udham Singh Nagar | **4** | 3 | **2** | 0 | **0** | 0 | **0** | 4 | 7 | 58.3 | Medium |
| Himachal Pradesh | Una | **4** | 3 | **1** | 0 | **0** | 0 | **0** | 4 | 7 | 58.3 | Medium |
| Arunachal Pradesh | Upper Siang | **4** | 3 | **2** | 0 | **0** | 0 | **0** | 4 | 7 | 58.3 | Medium |
| Uttarakhand | Uttarkashi | **4** | 3 | **2** | 0 | **0** | 0 | **0** | 4 | 7 | 58.3 | Medium |
| Arunachal Pradesh | West Kameng | **4** | 3 | **2** | 0 | **0** | 0 | **0** | 4 | 7 | 58.3 | Medium |
| Arunachal Pradesh | West Siang | **4** | 3 | **2** | 0 | **0** | 0 | **0** | 4 | 7 | 58.3 | Medium |
| Sikkim | West Sikkim | **4** | 3 | **1** | 0 | **0** | 0 | **0** | 4 | 7 | 58.3 | Medium |
| Nagaland | Wokha | **4** | 3 | **2** | 0 | **0** | 0 | **0** | 4 | 7 | 58.3 | Medium |
| Nagaland | Zunheboto | **4** | 3 | **2** | 0 | **0** | 0 | **0** | 4 | 7 | 58.3 | Medium |
| Uttar Pradesh | Amroha | **2** | 0 | **4** | 1 | **1** | 4 | **NA** | 1 | 6 | 50 | Medium |
| Madra Pradesh | Ashoknagar | **2** | 0 | **4** | 1 | **1** | 4 | **NA** | 1 | 6 | 50 | Medium |
| Rajasthan | Bhilwara | **1** | 0 | **3** | 1 | **1** | 4 | **NA** | 1 | 6 | 50 | Medium |
| Madra Pradesh | Bhopal | **3** | 1 | **2** | 0 | **1** | 4 | **NA** | 1 | 6 | 50 | Medium |
| Rajasthan | Bundi | **1** | 0 | **3** | 1 | **1** | 4 | **NA** | 1 | 6 | 50 | Medium |
| West Bengal | Darjeeling | **3** | 1 | **2** | 0 | **1** | 4 | **NA** | 1 | 6 | 50 | Medium |
| Rajasthan | Dausa | **1** | 0 | **3** | 1 | **1** | 4 | **NA** | 1 | 6 | 50 | Medium |
| Thripura | Dhalai | **3** | 1 | **3** | 1 | **0** | 0 | **0** | 4 | 6 | 50 | Medium |
| Mehalaya | East Garo Hills | **3** | 1 | **4** | 1 | **0** | 0 | **0** | 4 | 6 | 50 | Medium |
| Madra Pradesh | Guna | **2** | 0 | **4** | 1 | **1** | 4 | **NA** | 1 | 6 | 50 | Medium |
| Madra Pradesh | Harda | **2** | 0 | **3** | 1 | **1** | 4 | **NA** | 1 | 6 | 50 | Medium |
| Madra Pradesh | Hoshangabad | **2** | 0 | **3** | 1 | **1** | 4 | **NA** | 1 | 6 | 50 | Medium |
| Rajasthan | Jodhpur | **1** | 0 | **3** | 1 | **1** | 4 | **NA** | 1 | 6 | 50 | Medium |
| Rajasthan | Karauli | **1** | 0 | **4** | 1 | **1** | 4 | **NA** | 1 | 6 | 50 | Medium |
| Arunachal Pradesh | Lohit | **3** | 1 | **3** | 1 | **0** | 0 | **0** | 4 | 6 | 50 | Medium |
| Nagaland | Longleng | **3** | 1 | **3** | 1 | **0** | 0 | **0** | 4 | 6 | 50 | Medium |
| Telengana | Mahbubnagar | **1** | 0 | **3** | 1 | **1** | 4 | **NA** | 1 | 6 | 50 | Medium |
| Mizoram | Mamit | **3** | 1 | **3** | 1 | **0** | 0 | **0** | 4 | 6 | 50 | Medium |
| Nagaland | Mon | **3** | 1 | **3** | 1 | **0** | 0 | **0** | 4 | 6 | 50 | Medium |
| Telengana | Nizamabad | **3** | 1 | **2** | 0 | **1** | 4 | **NA** | 1 | 6 | 50 | Medium |
| Mehalaya | North Garo Hills | **3** | 1 | **3** | 1 | **0** | 0 | **0** | 4 | 6 | 50 | Medium |
| Thripura | North Tripura | **3** | 1 | **3** | 1 | **0** | 0 | **0** | 4 | 6 | 50 | Medium |
| Madra Pradesh | Rajagarh | **2** | 0 | **4** | 1 | **1** | 4 | **NA** | 1 | 6 | 50 | Medium |
| Rajasthan | Rajasmand | **1** | 0 | **3** | 1 | **1** | 4 | **NA** | 1 | 6 | 50 | Medium |
| Mehalaya | Ri-Bhoi | **3** | 1 | **4** | 1 | **0** | 0 | **0** | 4 | 6 | 50 | Medium |
| Bihar | KOSHI | **4** | 3 | **4** | 1 | **NA** | 1 | **NA** | 1 | 6 | 50 | Medium |
| Madra Pradesh | Shajapur | **2** | 0 | **3** | 1 | **1** | 4 | **NA** | 1 | 6 | 50 | Medium |
| Mehalaya | South West Garo Hills | **3** | 1 | **3** | 1 | **0** | 0 | **0** | 4 | 6 | 50 | Medium |
| Mehalaya | South West Khasi Hills | **3** | 1 | **3** | 1 | **0** | 0 | **0** | 4 | 6 | 50 | Medium |
| Bihar | SUPOL | **4** | 3 | **4** | 1 | **NA** | 1 | **NA** | 1 | 6 | 50 | Medium |
| Arunachal Pradesh | Tawang Town | **3** | 1 | **3** | 1 | **0** | 0 | **0** | 4 | 6 | 50 | Medium |
| Arunachal Pradesh | Tirap | **3** | 1 | **3** | 1 | **0** | 0 | **0** | 4 | 6 | 50 | Medium |
| Madra Pradesh | Vidisha | **2** | 0 | **4** | 1 | **1** | 4 | **NA** | 1 | 6 | 50 | Medium |
| Mehalaya | West Garo Hills | **3** | 1 | **3** | 1 | **0** | 0 | **0** | 4 | 6 | 50 | Medium |
| Mehalaya | West Khasi Hills | **3** | 1 | **3** | 1 | **0** | 0 | **0** | 4 | 6 | 50 | Medium |
| Uttarakhand | Dehradun | **3** | 1 | **2** | 0 | **0** | 0 | **0** | 4 | 5 | 41.7 | Low |
| Uttarakhand | Nainital | **3** | 1 | **2** | 0 | **0** | 0 | **0** | 4 | 5 | 41.7 | Low |
| Punjab | Pathankot | **3** | 1 | **1** | 0 | **0** | 0 | **0** | 4 | 5 | 41.7 | Low |
| Andra Pradesh | Ananthpur | **1** | 0 | **2** | 0 | **1** | 4 | **NA** | 1 | 5 | 41.7 | Low |
| Chhattisgarh | Balrampur | **4** | 3 | **4** | 1 | **0** | 0 | **NA** | 1 | 5 | 41.7 | Low |
| Chhattisgarh | Bastar | **4** | 3 | **4** | 1 | **0** | 0 | **NA** | 1 | 5 | 41.7 | Low |
| Chhattisgarh | Bijapur | **4** | 3 | **4** | 1 | **0** | 0 | **NA** | 1 | 5 | 41.7 | Low |
| Himachal Pradesh | Chamba | **3** | 1 | **1** | 0 | **0** | 0 | **0** | 4 | 5 | 41.7 | Low |
| Chhattisgarh | Dantewada | **4** | 3 | **4** | 1 | **0** | 0 | **NA** | 1 | 5 | 41.7 | Low |
| Assam | Darrang | **4** | 3 | **4** | 1 | **0** | 0 | **NA** | 1 | 5 | 41.7 | Low |
| Arunachal Pradesh | Upper Dibang Valley | **3** | 1 | **2** | 0 | **0** | 0 | **0** | 4 | 5 | 41.7 | Low |
| Assam | Dima Hasao | **4** | 3 | **3** | 1 | **0** | 0 | **NA** | 1 | 5 | 41.7 | Low |
| Mehalaya | East Khasi Hills | **3** | 1 | **2** | 0 | **0** | 0 | **0** | 4 | 5 | 41.7 | Low |
| Thripura | Gomati | **3** | 1 | **2** | 0 | **0** | 0 | **0** | 4 | 5 | 41.7 | Low |
| Himachal Pradesh | Kangra | **3** | 1 | **1** | 0 | **0** | 0 | **0** | 4 | 5 | 41.7 | Low |
| Assam | Karbi Anglong | **4** | 3 | **4** | 1 | **0** | 0 | **NA** | 1 | 5 | 41.7 | Low |
| Assam | West Karbi Anglong | **4** | 3 | **4** | 1 | **0** | 0 | **NA** | 1 | 5 | 41.7 | Low |
| Thripura | Khowai | **3** | 1 | **2** | 0 | **0** | 0 | **0** | 4 | 5 | 41.7 | Low |
| Himachal Pradesh | Kinnaur | **3** | 1 | **1** | 0 | **0** | 0 | **0** | 4 | 5 | 41.7 | Low |
| Mizoram | Kolasib | **3** | 1 | **1** | 0 | **0** | 0 | **0** | 4 | 5 | 41.7 | Low |
| Chhattisgarh | Korba | **4** | 3 | **3** | 1 | **0** | 0 | **NA** | 1 | 5 | 41.7 | Low |
| Himachal Pradesh | Kullu | **3** | 1 | **1** | 0 | **0** | 0 | **0** | 4 | 5 | 41.7 | Low |
| Andra Pradesh | Kurnool | **1** | 0 | **2** | 0 | **1** | 4 | **NA** | 1 | 5 | 41.7 | Low |
| Himachal Pradesh | Lahaul and Spiti | **3** | 1 | **1** | 0 | **0** | 0 | **0** | 4 | 5 | 41.7 | Low |
| Mizoram | Lawngtlai | **3** | 1 | **2** | 0 | **0** | 0 | **0** | 4 | 5 | 41.7 | Low |
| Mizoram | Lunglei | **3** | 1 | **1** | 0 | **0** | 0 | **0** | 4 | 5 | 41.7 | Low |
| Telengana | Medak | **2** | 0 | **2** | 0 | **1** | 4 | **NA** | 1 | 5 | 41.7 | Low |
| Haryana | Mewat | **1** | 0 | **4** | 1 | **0** | 0 | **0** | 4 | 5 | 41.7 | Low |
| Assam | Hojai | **4** | 3 | **3** | 1 | **0** | 0 | **NA** | 1 | 5 | 41.7 | Low |
| Assam | Nagaon | **4** | 3 | **3** | 1 | **0** | 0 | **NA** | 1 | 5 | 41.7 | Low |
| Telengana | Nalgonda | **1** | 0 | **2** | 0 | **1** | 4 | **NA** | 1 | 5 | 41.7 | Low |
| Chhattisgarh | Narayanpur | **4** | 3 | **4** | 1 | **0** | 0 | **NA** | 1 | 5 | 41.7 | Low |
| Maharashtra | Nashik | **2** | 0 | **2** | 0 | **1** | 4 | **NA** | 1 | 5 | 41.7 | Low |
| Goa | North Goa | **3** | 1 | **1** | 0 | **0** | 0 | **0** | 4 | 5 | 41.7 | Low |
| Sikkim | North Sikkim | **3** | 1 | **1** | 0 | **0** | 0 | **0** | 4 | 5 | 41.7 | Low |
| Odisha | Nuapada | **4** | 3 | **4** | 1 | **NA** | 1 | **1** | 0 | 5 | 41.7 | Low |
| Chhattisgarh | Raipur | **3** | 1 | **2** | 0 | **1** | 4 | **1** | 0 | 5 | 41.7 | Low |
| Telengana | Rangareddi | **2** | 0 | **2** | 0 | **1** | 4 | **NA** | 1 | 5 | 41.7 | Low |
| Punjab | Rupnagar | **3** | 1 | **1** | 0 | **0** | 0 | **0** | 4 | 5 | 41.7 | Low |
| Mizoram | Saiha | **3** | 1 | **1** | 0 | **0** | 0 | **0** | 4 | 5 | 41.7 | Low |
| Maharashtra | Satara | **2** | 0 | **2** | 0 | **1** | 4 | **NA** | 1 | 5 | 41.7 | Low |
| Thripura | Sipahijala | **3** | 1 | **2** | 0 | **0** | 0 | **0** | 4 | 5 | 41.7 | Low |
| Mehalaya | South Garo Hills | **3** | 1 | **2** | 0 | **0** | 0 | **0** | 4 | 5 | 41.7 | Low |
| Goa | South Goa | **3** | 1 | **1** | 0 | **0** | 0 | **0** | 4 | 5 | 41.7 | Low |
| Thripura | South Tripura | **3** | 1 | **2** | 0 | **0** | 0 | **0** | 4 | 5 | 41.7 | Low |
| Thripura | Unakoti | **3** | 1 | **2** | 0 | **0** | 0 | **0** | 4 | 5 | 41.7 | Low |
| Thripura | West Tripura | **3** | 1 | **1** | 0 | **0** | 0 | **0** | 4 | 5 | 41.7 | Low |
| Andra Pradesh | Kadapa | **1** | 0 | **2** | 0 | **1** | 4 | **NA** | 1 | 5 | 41.7 | Low |
| Haryana | Yamunanagar | **3** | 1 | **1** | 0 | **0** | 0 | **0** | 4 | 5 | 41.7 | Low |
| Chhattisgarh | Balod | **4** | 3 | **3** | 1 | **0** | 0 | **NA** | 1 | 5 | 41.7 | Low |
| Chhattisgarh | Gariaband | **4** | 3 | **3** | 1 | **0** | 0 | **NA** | 1 | 5 | 41.7 | Low |
| Odisha | Jagatsinghpur | **4** | 3 | **3** | 1 | **NA** | 1 | **1** | 0 | 5 | 41.7 | Low |
| Chhattisgarh | Kondagaon | **4** | 3 | **3** | 1 | **0** | 0 | **NA** | 1 | 5 | 41.7 | Low |
| Chhattisgarh | Koriya | **4** | 3 | **3** | 1 | **0** | 0 | **NA** | 1 | 5 | 41.7 | Low |
| Assam | Morigaon | **4** | 3 | **3** | 1 | **0** | 0 | **NA** | 1 | 5 | 41.7 | Low |
| Chhattisgarh | Rajnandgaon | **4** | 3 | **3** | 1 | **0** | 0 | **NA** | 1 | 5 | 41.7 | Low |
| Chhattisgarh | Sukma | **4** | 3 | **3** | 1 | **0** | 0 | **NA** | 1 | 5 | 41.7 | Low |
| Chhattisgarh | Surajpur | **4** | 3 | **3** | 1 | **0** | 0 | **NA** | 1 | 5 | 41.7 | Low |
| Chhattisgarh | Kanker | **4** | 3 | **3** | 1 | **0** | 0 | **NA** | 1 | 5 | 41.7 | Low |
| Punjab | Fazilka | **1** | 0 | **1** | 0 | **0** | 0 | **0** | 4 | 4 | 33.3 | Low |
| Haryana | Ambala | **2** | 0 | **1** | 0 | **0** | 0 | **0** | 4 | 4 | 33.3 | Low |
| Punjab | Amritsar | **1** | 0 | **1** | 0 | **0** | 0 | **0** | 4 | 4 | 33.3 | Low |
| Punjab | Barnala | **1** | 0 | **1** | 0 | **0** | 0 | **0** | 4 | 4 | 33.3 | Low |
| Punjab | Bathinda | **1** | 0 | **1** | 0 | **0** | 0 | **0** | 4 | 4 | 33.3 | Low |
| Haryana | Bhiwani | **1** | 0 | **2** | 0 | **0** | 0 | **0** | 4 | 4 | 33.3 | Low |
| Haryana | Charkhi Dadr | **1** | 0 | **2** | 0 | **0** | 0 | **0** | 4 | 4 | 33.3 | Low |
| Andra Pradesh | Chittoor | **2** | 0 | **2** | 0 | **1** | 4 | **1** | 0 | 4 | 33.3 | Low |
| Haryana | Faridabad | **1** | 0 | **1** | 0 | **0** | 0 | **0** | 4 | 4 | 33.3 | Low |
| Punjab | Faridkot | **1** | 0 | **1** | 0 | **0** | 0 | **0** | 4 | 4 | 33.3 | Low |
| Haryana | Fatehabad | **1** | 0 | **1** | 0 | **0** | 0 | **0** | 4 | 4 | 33.3 | Low |
| Punjab | Fatehgarh Sahib | **1** | 0 | **1** | 0 | **0** | 0 | **0** | 4 | 4 | 33.3 | Low |
| Punjab | Firozpur | **1** | 0 | **2** | 0 | **0** | 0 | **0** | 4 | 4 | 33.3 | Low |
| Punjab | Gurdaspur | **1** | 0 | **1** | 0 | **0** | 0 | **0** | 4 | 4 | 33.3 | Low |
| Haryana | Gurgaon | **1** | 0 | **1** | 0 | **0** | 0 | **0** | 4 | 4 | 33.3 | Low |
| Haryana | Hisar | **1** | 0 | **1** | 0 | **0** | 0 | **0** | 4 | 4 | 33.3 | Low |
| Punjab | Hoshiarpur | **2** | 0 | **1** | 0 | **0** | 0 | **0** | 4 | 4 | 33.3 | Low |
| Punjab | Jalandhar | **1** | 0 | **1** | 0 | **0** | 0 | **0** | 4 | 4 | 33.3 | Low |
| Chhattisgarh | Jashpur | **4** | 3 | **4** | 1 | **0** | 0 | **1** | 0 | 4 | 33.3 | Low |
| Haryana | Jhajjar | **1** | 0 | **1** | 0 | **0** | 0 | **0** | 4 | 4 | 33.3 | Low |
| Uttar Pradesh | Jhansi | **1** | 0 | **2** | 0 | **0** | 0 | **0** | 4 | 4 | 33.3 | Low |
| Haryana | Jind | **1** | 0 | **1** | 0 | **0** | 0 | **0** | 4 | 4 | 33.3 | Low |
| Haryana | Kaithal | **1** | 0 | **1** | 0 | **0** | 0 | **0** | 4 | 4 | 33.3 | Low |
| Assam | Kamrup Metro. | **4** | 3 | **2** | 0 | **0** | 0 | **NA** | 1 | 4 | 33.3 | Low |
| Punjab | Kapurthala | **1** | 0 | **1** | 0 | **0** | 0 | **0** | 4 | 4 | 33.3 | Low |
| Haryana | Karnal | **1** | 0 | **1** | 0 | **0** | 0 | **0** | 4 | 4 | 33.3 | Low |
| Odisha | Khordha | **4** | 3 | **2** | 0 | **NA** | 1 | **1** | 0 | 4 | 33.3 | Low |
| Haryana | Kurukshetra | **1** | 0 | **1** | 0 | **0** | 0 | **0** | 4 | 4 | 33.3 | Low |
| Uttar Pradesh | Lalitpur | **3** | 1 | **3** | 1 | **NA** | 1 | **NA** | 1 | 4 | 33.3 | Low |
| Punjab | Ludhiana | **1** | 0 | **1** | 0 | **0** | 0 | **0** | 4 | 4 | 33.3 | Low |
| Chhattisgarh | Mahasamund | **4** | 3 | **3** | 1 | **0** | 0 | **1** | 0 | 4 | 33.3 | Low |
| Haryana | Mahendragarh | **1** | 0 | **1** | 0 | **0** | 0 | **0** | 4 | 4 | 33.3 | Low |
| Punjab | Mansa | **1** | 0 | **1** | 0 | **0** | 0 | **0** | 4 | 4 | 33.3 | Low |
| Punjab | Moga | **1** | 0 | **1** | 0 | **0** | 0 | **0** | 4 | 4 | 33.3 | Low |
| Uttar Pradesh | Moradabad | **3** | 1 | **3** | 1 | **NA** | 1 | **NA** | 1 | 4 | 33.3 | Low |
| Punjab | Muktsar | **1** | 0 | **1** | 0 | **0** | 0 | **0** | 4 | 4 | 33.3 | Low |
| Haryana | Palwal | **1** | 0 | **2** | 0 | **0** | 0 | **0** | 4 | 4 | 33.3 | Low |
| Haryana | Panipat | **1** | 0 | **1** | 0 | **0** | 0 | **0** | 4 | 4 | 33.3 | Low |
| Punjab | Patiala | **1** | 0 | **1** | 0 | **0** | 0 | **0** | 4 | 4 | 33.3 | Low |
| Andra Pradesh | Prakasam | **1** | 0 | **2** | 0 | **1** | 4 | **1** | 0 | 4 | 33.3 | Low |
| Odisha | Puri | **4** | 3 | **2** | 0 | **NA** | 1 | **1** | 0 | 4 | 33.3 | Low |
| Chhattisgarh | Raigarh | **4** | 3 | **3** | 1 | **0** | 0 | **1** | 0 | 4 | 33.3 | Low |
| Haryana | Rewari | **1** | 0 | **1** | 0 | **0** | 0 | **0** | 4 | 4 | 33.3 | Low |
| Haryana | Rohtak | **1** | 0 | **1** | 0 | **0** | 0 | **0** | 4 | 4 | 33.3 | Low |
| Punjab | Mohali | **1** | 0 | **1** | 0 | **0** | 0 | **0** | 4 | 4 | 33.3 | Low |
| Punjab | Sangrur | **1** | 0 | **1** | 0 | **0** | 0 | **0** | 4 | 4 | 33.3 | Low |
| Punjab | Shahid Bhagat Singh Nagar | **1** | 0 | **1** | 0 | **0** | 0 | **0** | 4 | 4 | 33.3 | Low |
| Haryana | Sirsa | **1** | 0 | **2** | 0 | **0** | 0 | **0** | 4 | 4 | 33.3 | Low |
| Haryana | Sonipat | **1** | 0 | **1** | 0 | **0** | 0 | **0** | 4 | 4 | 33.3 | Low |
| Chhattisgarh | Surguja | **4** | 3 | **4** | 1 | **0** | 0 | **1** | 0 | 4 | 33.3 | Low |
| Punjab | Tarn Taran | **1** | 0 | **1** | 0 | **0** | 0 | **0** | 4 | 4 | 33.3 | Low |
| Assam | Barpeta | **3** | 1 | **3** | 1 | **0** | 0 | **NA** | 1 | 3 | 25 | Low |
| Assam | Bongaigaon | **3** | 1 | **3** | 1 | **0** | 0 | **NA** | 1 | 3 | 25 | Low |
| Assam | Cachar | **3** | 1 | **4** | 1 | **0** | 0 | **NA** | 1 | 3 | 25 | Low |
| Assam | Chirang | **3** | 1 | **3** | 1 | **0** | 0 | **NA** | 1 | 3 | 25 | Low |
| Chhattisgarh | Dhamtari | **4** | 3 | **2** | 0 | **0** | 0 | **1** | 0 | 3 | 25 | Low |
| Assam | Dhemaji | **3** | 1 | **3** | 1 | **0** | 0 | **NA** | 1 | 3 | 25 | Low |
| Assam | South Salmara-Mankachar | **3** | 1 | **4** | 1 | **0** | 0 | **NA** | 1 | 3 | 25 | Low |
| Assam | Dhubri | **3** | 1 | **4** | 1 | **0** | 0 | **NA** | 1 | 3 | 25 | Low |
| Assam | Dibrugarh | **3** | 1 | **3** | 1 | **0** | 0 | **NA** | 1 | 3 | 25 | Low |
| Assam | Goalpara | **3** | 1 | **4** | 1 | **0** | 0 | **NA** | 1 | 3 | 25 | Low |
| Assam | Hailakandi | **3** | 1 | **4** | 1 | **0** | 0 | **NA** | 1 | 3 | 25 | Low |
| Chhattisgarh | Kabirdham | **3** | 1 | **4** | 1 | **0** | 0 | **NA** | 1 | 3 | 25 | Low |
| Assam | Karimganj | **3** | 1 | **4** | 1 | **0** | 0 | **NA** | 1 | 3 | 25 | Low |
| Assam | Kokrajhar | **3** | 1 | **3** | 1 | **0** | 0 | **NA** | 1 | 3 | 25 | Low |
| Assam | Lakhimpur | **3** | 1 | **3** | 1 | **0** | 0 | **NA** | 1 | 3 | 25 | Low |
| Assam | Biswanath | **3** | 1 | **3** | 1 | **0** | 0 | **NA** | 1 | 3 | 25 | Low |
| Assam | Sonitpur | **3** | 1 | **3** | 1 | **0** | 0 | **NA** | 1 | 3 | 25 | Low |
| Assam | Tinsukia | **3** | 1 | **4** | 1 | **0** | 0 | **NA** | 1 | 3 | 25 | Low |
| Assam | Udalguri | **3** | 1 | **3** | 1 | **0** | 0 | **NA** | 1 | 3 | 25 | Low |
| Chhattisgarh | Baloda Bazar | **3** | 1 | **3** | 1 | **0** | 0 | **NA** | 1 | 3 | 25 | Low |
| Chhattisgarh | Bemetara. | **3** | 1 | **3** | 1 | **0** | 0 | **NA** | 1 | 3 | 25 | Low |
| Chhattisgarh | Mungeli | **3** | 1 | **3** | 1 | **0** | 0 | **NA** | 1 | 3 | 25 | Low |
| Assam | Sivasagar | **3** | 1 | **3** | 1 | **0** | 0 | **NA** | 1 | 3 | 25 | Low |
| Assam | Charaideo | **3** | 1 | **3** | 1 | **0** | 0 | **NA** | 1 | 3 | 25 | Low |
| Assam | Baksa | **3** | 1 | **2** | 0 | **0** | 0 | **NA** | 1 | 2 | 16.7 | Very low |
| Chhattisgarh | Bilaspur | **3** | 1 | **3** | 1 | **0** | 0 | **1** | 0 | 2 | 16.7 | Very low |
| Assam | Golaghat | **3** | 1 | **2** | 0 | **0** | 0 | **NA** | 1 | 2 | 16.7 | Very low |
| Chhattisgarh | Janjgir-Champa | **3** | 1 | **3** | 1 | **0** | 0 | **1** | 0 | 2 | 16.7 | Very low |
| Assam | Jorhat | **3** | 1 | **2** | 0 | **0** | 0 | **NA** | 1 | 2 | 16.7 | Very low |
| Assam | Majuli | **3** | 1 | **2** | 0 | **0** | 0 | **NA** | 1 | 2 | 16.7 | Very low |
| Assam | Kamrup | **3** | 1 | **2** | 0 | **0** | 0 | **NA** | 1 | 2 | 16.7 | Very low |
| Assam | Nalbari | **3** | 1 | **2** | 0 | **0** | 0 | **NA** | 1 | 2 | 16.7 | Very low |
| Chhattisgarh | Durg | **3** | 1 | **2** | 0 | **0** | 0 | **1** | 0 | 1 | 8.33 | Very low |

Districts shown in the table are those identified by State Representatives engaged through the Consultative Workshop on Podoconiosis held in Kochi, December 2019.

1. Environmental suitability- quartiles of suitability based on extrapolation of the environmental niche of podoconiosis characterised using data from prevalence surveys in Africa.
2. Poverty- quartiles of multidimensional poverty (MDPI) modelled by the Oxford Poverty and Human Development Initiative [1].
3. Lymphoedema cases within the district known to state health authorities, according to state health representatives.
4. MMDP Services assumed to be provided based on the coverage of the National Lymphatic Filariasis Elimination Programme, according to state health representatives.
5. Consensus Score- sum of scores assigned for each of the above four components.

References:

1. Alkire S, Oldiges, C. and Kanagaratnam, U. Multidimensional poverty reduction in India 2005/6–2015/16: still a long way to go but the poorest are catching up. OPHI Research in Progress 54a, University of Oxford2018.
