# Supplementary material for: Developing consensus of evidence to target case finding surveys for podoconiosis: a potentially forgotten disease in India
Source: Trans R Soc Trop Med Hyg. 2020 Nov 9;114(12):908–15. doi: 10.1093/trstmh/traa064 (PMC7738658; doi:10.1093/trstmh/traa064)
Supplement: traa064_Supplemental_Files [file traa064_supplemental_files.zip › Supplementary Figure 2.pdf]

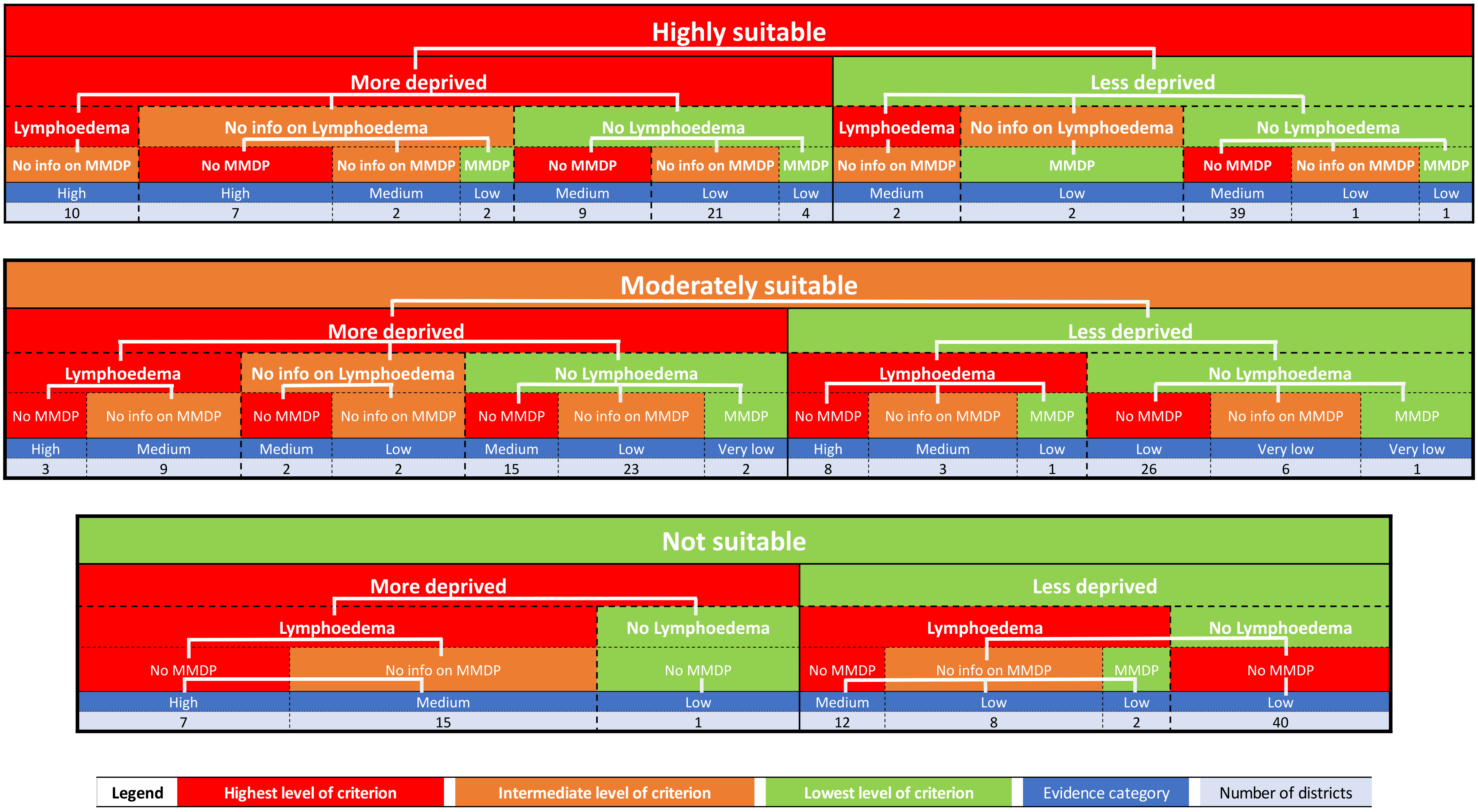

Highly suitable
More deprived
Less deprived
Lymphoedema
No info on Lymphoedema
No Lymphoedema
Lymphoedema
No info on Lymphoedema
No Lymphoedema
No info on MMDP
No MMDP
No info on MMDP
MMDP
No MMDP
No info on MMDP
MMDP
No info on MMDP
MMDP
No MMDP
No info on MMDP
MMDP
High
High
Medium
Low
Medium
Low
Low
Medium
Low
Medium
Low
Low
10
7
2
2
9
21
4
2
2
39
1
1
Moderately suitable
More deprived
Less deprived
Lymphoedema
No info on Lymphoedema
No Lymphoedema
Lymphoedema
No Lymphoedema
No MMDP
No info on MMDP
No MMDP
No info on MMDP
No MMDP
No info on MMDP
MMDP
No MMDP
No info on MMDP
MMDP
No MMDP
No info on MMDP
MMDP
High
Medium
Medium
Low
Medium
Low
Very low
High
Medium
Low
Low
Very low
Very low
3
9
2
2
15
23
2
8
3
1
26
6
1
Not suitable
More deprived
Less deprived
Lymphoedema
No Lymphoedema
Lymphoedema
No Lymphoedema
No MMDP
No info on MMDP
No MMDP
No MMDP
No info on MMDP
MMDP
No MMDP
High
Medium
Low
Medium
Low
Low
Low
7
15
1
12
8
2
40
Legend
Highest level of criterion
Intermediate level of criterion
Lowest level of criterion
Evidence category
Number of districts
