# Supplementary material for: Developing consensus of evidence to target case finding surveys for podoconiosis: a potentially forgotten disease in India
Source: Trans R Soc Trop Med Hyg. 2020 Nov 9;114(12):908–15. doi: 10.1093/trstmh/traa064 (PMC7738658; doi:10.1093/trstmh/traa064)
Supplement: traa064_Supplemental_Files [file traa064_supplemental_files.zip › Supplementary Methods.docx]

S1. Methods: Linkage of data to shapefile for mapping

Due to redistricting and differences in the uptake of new district configurations between states, some of the districts described by state representatives corresponded to districts in the GADM shapefile from 2015 while others corresponded to the shapefile representing districts in 2012.

Districts represented in 2015 were linked directly to the 2015 shapefile based on the state and district name while districts represented in 2012 were linked to the 2015 shapefile using spatial tools implemented in R. The two shapefiles were overlaid and the proportion of the area of each of the 2012 districts overlapping with a district from 2015 was calculated. Each of the 2012 districts was linked to the 2015 district that contained the greatest proportion of its area. This link was used to join the districts listed in the workshop to those represented by the 2015 shapefile.

In instances where districts had been merged in 2015 to create a single new district, the evidence from all constituent districts described in the workshop was combined to represent the evidence in the new district. For instance, if participants indicated evidence of lymphedema cases in a district which was merged to one that didn’t, the new district was considered to have evidence of lymphedema cases. If participants indicated that MDA was done in one district which was merged with another with no MDA, the new district was considered to implement MDA.
