# Supplementary figures and images for: Developing consensus of evidence to target case finding surveys for podoconiosis: a potentially forgotten disease in India
Source: Trans R Soc Trop Med Hyg. 2020 Nov 9;114(12):908–15. doi: 10.1093/trstmh/traa064 (PMC7738658; doi:10.1093/trstmh/traa064)

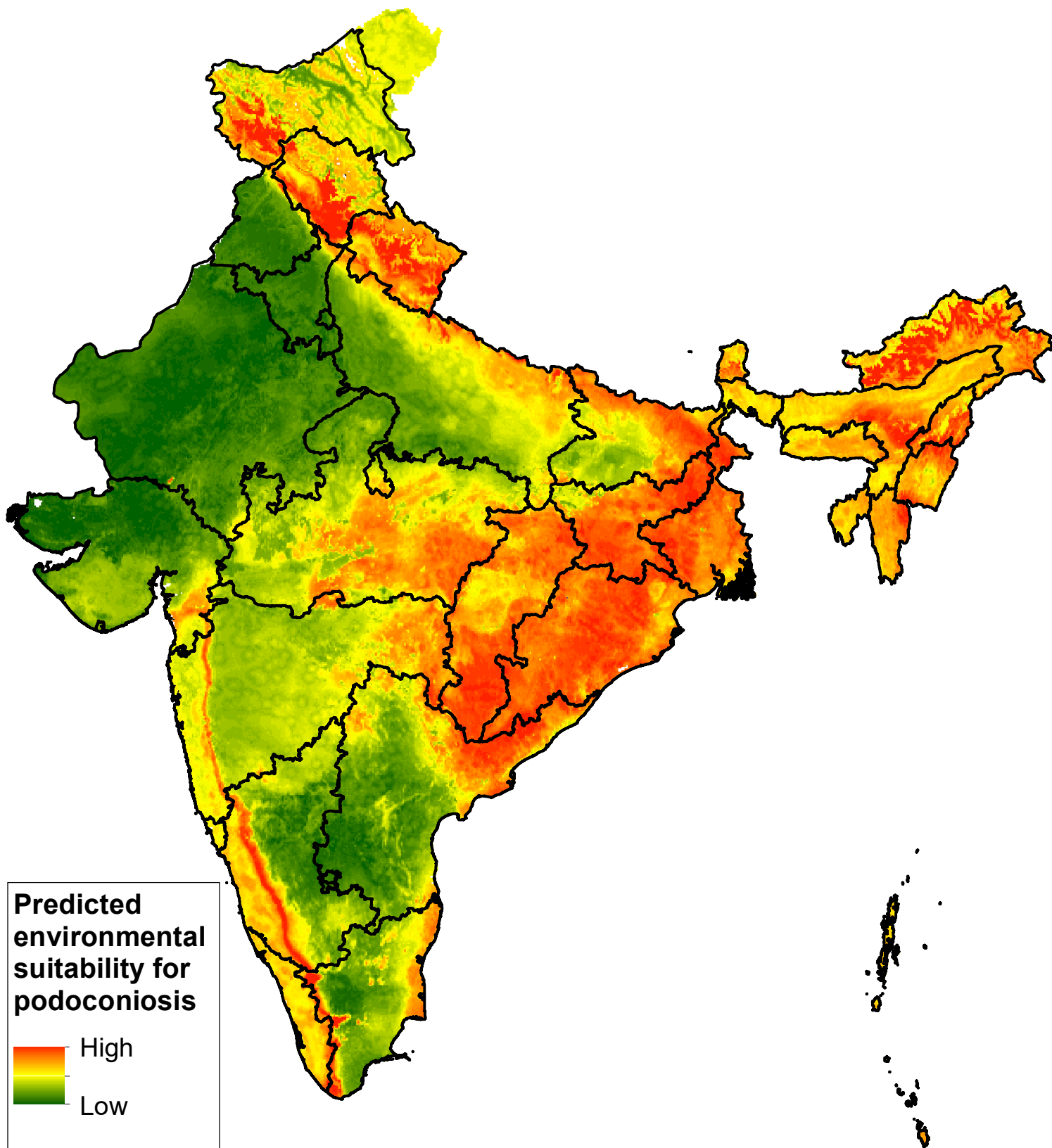

Supplement: traa064_Supplemental_Files [file traa064_supplemental_files.zip › Supplementary Figure 1.pdf]
